# Effects of compound stimulation of fluid shear stress plus ultrasound on stem cell proliferation and osteogenesis

**DOI:** 10.1093/rb/rbab066

**Published:** 2021-11-18

**Authors:** Lingzhi Jing, Suna Fan, Xiang Yao, Yaopeng Zhang

**Affiliations:** 1 State Key Laboratory for Modification of Chemical Fibers and Polymer Materials, Shanghai Engineering Research Center of Nano-Biomaterials and Regenerative Medicine, College of Materials Science and Engineering, Donghua University, Shanghai 201620, P.R. China; 2 Jinan Jinquan Bio-Technology Co. Ltd, Jinan 250101, P.R. China

**Keywords:** compound stimulation, microfluidic chip, stem cell, cell proliferation, cell differentiation

## Abstract

Bone tissue with strong adaptability is often in a complex dynamical microenvironment *in vivo*, which is associated with the pathogenesis and treatment of orthopedic diseases. Therefore, it is of great significance to investigate the effects of corresponding compound stimulation on cell behaviors. Herein, a fluid shear stress (FSS) plus ultrasound stimulation platform suitable for cell studies based on a microfluidic chip was constructed and bone marrow mesenchymal stem cell (BMSC) was chosen as a model cell. The proliferation and osteogenesis of BMSCs under the compound stimulation of FSS plus ultrasound in growth medium without any soluble induction factors were firstly investigated. Single FSS stimulation and static culture conditions were also examined. Results illustrated that suitable single FSS stimulation (about 0.06 dyn/cm^2^) could significantly enhance cell proliferation and osteogenesis simultaneously when compared to the static control, while greater FSS mitigated or even restricted these enhancing effects. Interestingly, ultrasound stimulation combined with this suitable FSS stimulation further accelerated cell proliferation as the intensity of ultrasound increasing. As for the osteogenesis under compound stimulation, it was relatively restricted under lower ultrasound intensity (about 0.075 W/cm^2^), while promoted when the intensity became higher (about 1.75 W/cm^2^). This study suggests that both the cell proliferation and osteogenesis are very responsive to the magnitudes of FSS and ultrasound stimulations and can be both significantly enhanced by proper combination strategies. Moreover, these findings will provide valuable references for the construction of effective cell bioreactors and also the treatment of orthopedic diseases.

## Introduction

Many factors, such as congenital defects or acquired injuries can lead to bone tissue disease or defect, which will seriously affect the living qualities of patients. With the pluripotent capability, stem cell, especially bone marrow mesenchymal stem cells (BMSCs), combined with proper tissue scaffolds, has become a promising tissue engineering strategy for the treatment of bone and cartilage defects [1–9]. In order to meet the clinical treatment demands, on the one hand, it is necessary to obtain a sufficient number of seeding cells, on the other hand, it is also very important to regulate stem cell differentiation toward required direction. Therefore, it is of great significance to study and reveal the crucial factors which can effectively regulate cell proliferation and differentiation.

BMSCs, located in the bone marrow cavity, are capable to differentiate into osteoblast [10–13], chondroblast [14–16], neuroblast [17, 18] and other cell lineages under appropriate conditions. Up to now, it is already widely reported that a variety of chemical and physical cues can affect the proliferation and differentiation of BMSCs, such as soluble factors [19–23], ligand distribution [24–26], substrate stiffness [27–29], surface chemical composition [30–33], topological structure [34–39], or even cell shapes [40–42]. Bone tissues *in vivo* are often in a complex dynamical microenvironment combined with various stimulations, such as fluid shear stress (FSS), compression stress and hydrostatic pressure caused by motions. So, cells in the bone marrow, such as BMSCs, will inevitably experience the relevant compound stimulation [43]. Therefore, it is of great value to investigate the effects of compound stimulation on cell behaviors [44, 45]. With the development of material science and biomechanical loading equipment, the influence of a few kinds of single mechanical stimulations on cell behaviors has gradually been revealed. For example, some studies have confirmed that single tensile stress [46, 47], hydrostatic pressure [48, 49] or FSS [50, 51] *in vitro* could all significantly affect cell adhesion and differentiation, especially the chondrogenesis of stem cell [52–55].

Ultrasound is described as mechanical sonic waves with a frequency higher than the upper limit of human hearing. As you may know, it is usually used as a safe and noninvasive diagnostic tool in clinical practice [56, 57]. Interestingly, it is also discovered that in addition to diagnosis, ultrasound can be effectively applied for disease treatment according to its category. For example, high-intensity focused ultrasound (HIFUS) can be used for the ablation of malignant tissues [58], and low-intensity pulsed ultrasound (LIPUS) can be used for the adjuvant therapy of bone diseases [59, 60].

**Figure 1.**
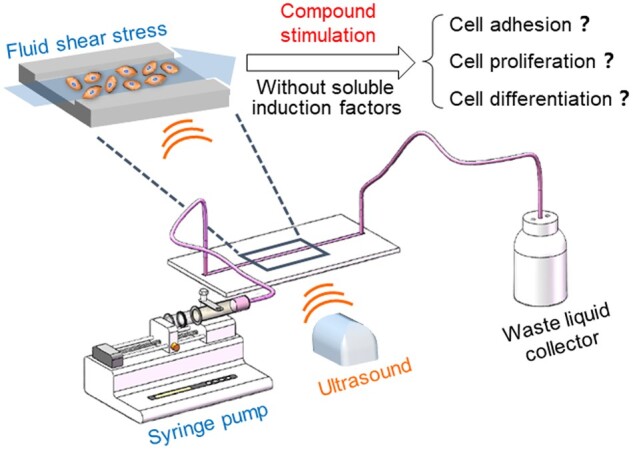
Schematic presentation of idea to explore the effects of compound stimulation (FSS combined with ultrasound) on cell behaviors

As mentioned above, the microenvironment of *in vivo* bone tissue is often complex and composed of various stimulations. The combination of multiple cues can simulate the complex microenvironment more effectively. However, there have been few reports that comprehensively examined the effects of compound stimulation on cell behaviors. In this study, rat BMSCs were chosen as a model cell. A FSS plus ultrasound stimulation platform suitable for cell studies was developed based on a microfluidic chip. The effects of FSS plus ultrasound compound stimulation on BMSCs adhesion, proliferation and osteogenic differentiation in growth medium without any soluble induction factors were firstly examined based on the developed cell culture platform, as schematically illustrated in Fig. 1.

In the designed cell culture platform, the FSS stimulation is generated by the flow of culture medium and can be adjusted by changing corresponding perfusion speed. The ultrasound stimulation is applied through an effective ultrasonic coupling agent between the bottom of microchannel and ultrasonic probe. The intensity of ultrasound stimulation can be adjusted by selecting different power level on the ultrasonic apparatus. Through the construction of compound stimulation platform and careful investigation of the influence of FSS plus ultrasound stimulation on BMSCs behaviors, it may afford valuable information for better understanding the effects of compound stimulation on cell behaviors and further provide useful guidance for the construction of effective cell bioreactors.

## Experiments and methods

### Materials

The Sylgard 184 poly(dimethylsiloxane) (PDMS) and curing agent were purchased from Dow Corning (USA). The photoresist mold with microchannel patterns was purchased from Suzhou CChip Scientific Instrument Co., Ltd. (China, the microchannel patterns were designed by ourselves). Fetal bovine serum (FBS), minimum essential medium α (α-MEM), Dulbecco’s modified Eagle’s medium (DMEM), penicillin, streptomycin and 0.25% trypsin-EDTA were purchased from Gibco (USA). The fibronectin was purchased from Roche (Switzerland). TRITC-phalloidin and 2-(4-amidinophenyl)-6-indolecarbamidine dihydrochloride (DAPI) were purchased from Sigma-Aldrich (USA). The BCIP/NBT alkaline phosphatase color development kit was purchased from Beyotime Biotechnology Co., Ltd. (China). The MagneSil^@^ Total RNA mini isolation system was purchased from Promega (USA). Primary rat BMSCs were purchased from Shanghai AllCells Biotech Co., Ltd. (China). The polytetrafluoroethylene (PTFE) tube, 1 mm in inner diameter and 1.6 mm in outer diameter, was bought from Suzhou Wenhao Microfluidics Technology Co., Ltd. (China).

### Construction of the compound stimulation platform based on microfluidic chips

The whole platform consists of three parts: a microfluidic chip for cell culture; a perfusion system based on an injection pump (KDS210P, KD Scientific) and corresponding PTFE tubes; an ultrasound system based on a LIPUS instrument (WBL-ED, Wanbeli Medical devices Co. Ltd.).

A replica of the designed microchannel was built by rapid prototyping of PDMS [61–63]. In brief, PDMS prepolymer was mixed with its curing agent at a ratio of 10:1 and then poured onto the photoresist mold with microchannel patterns. After curing at about 70°C for 1 h, the PDMS replica with microchannel patterns can be removed from the photoresist mold and then punched with 2 mm diameter hole puncher at the inlet and outlet of the channel. The smooth PDMS membrane was obtained by the similar procedure while using a flat substrate mold. After punching, the PDMS replica with microchannel patterns and another thin smooth PDMS membrane were exposed to 500 W oxygen plasma environment for 5 min, then bonded together to get the microfluidic chip. The FSS in the microchannel can be realized through the propulsion of cell culture medium. The inlet of the channel is connected to the syringe by PTFE tube, and the outlet is connected by PTFE tube to the waste liquid collector, as schematically shown in Fig. 1. The ultrasound stimulation was applied to the bottom of the chip through the ultrasonic probe. The probe coated with ultrasonic coupling agent is tightly glued to the bottom of the chip, conducting the ultrasound stimulation into the microchannel. The combination of FSS and ultrasound stimulations create a compound stimulation for the cells cultured in microchannel. The intensities of the FSS and ultrasound could be independently adjusted by controlling the propulsion rate of injection pump and the power of ultrasound instrument.

### Morphology observation of the microfluidic chip

Cross-sectional morphologies of the microchannel were observed using a scanning electron microscope (SEM, SU8010, Hitachi). Before observation, the samples were sputter-coated with gold under 15 mA for 20 s. In addition, typical morphological features of the microchannel have also been detected by a scanning white-light interferometry profilometer (WYKO/NT9100, Veeco).

### Cell culturing

The BMSCs were cultured in α-MEM with 10% FBS, 1% penicillin and 1% streptomycin at 37°C with 5% CO_2_ atmosphere in a humidified incubator. Cells were digested with 0.25% trypsin-EDTA and passaged at nearly 80% confluence. Only passages 2–3 of primary BMSCs were used in the later experiments.

### Cell seeding and culturing in the microchannels

In order to promote cell adhesion in the microchannel and hence increasing the intensity scope of investigated FSS, the inner surface of all the microchannels were coated with 25 μg/ml fibronectin solution for 12 h in the incubator after sterilization. Then the BMSCs with a concentration of 8 × 10^5^ cells/ml were injected into the channel at a constant speed of 50 ml/h through the injection pump until the cell suspension fill the whole channel. After incubation for 1 day, unattached cells were removed and fresh culture medium was injected into the channel. For cell culture in the microchannels, the culture medium was high-glucose DMEM mixed with 10% FBS.

For the cells cultured in static condition, the culture medium was changed every 2 days at a perfusion rate of 5 μl/min (about 5 min per time). For cells cultured in perfusion conditions (single FSS stimulation), they were cultured under continuous perfusion for 6 h per day with varied flow rates of 5, 15 and 45 μl/min, respectively.

For compound stimulation, the effective working area of the ultrasonic probe is about 10 cm^2^ and the power of combined ultrasound is 0.75, 5 and 17.5 W, respectively. More specific conditions were as follows: on the basis of proper perfusion culture (5 μl/min, 6 h/day), the above three ultrasound stimulations (10 min per day) with different intensities were combined at the perfusion time. Corresponding compound stimulation groups were denoted as I (combined with 0.075 W/cm^2^ ultrasound stimulation, the ultrasound intensity is calculated by ultrasound power divided by the effective working area of ultrasonic probe), II (combined with 0.5 W/cm^2^ ultrasound stimulation) and III (combined with 1.75 W/cm^2^ ultrasound stimulation), respectively.

### Cell staining

After 4 days of culture in the microchannels, all samples (static culture, single FSS stimulation and compound stimulation) were fixed with 4% paraformaldehyde for 15 min at room temperature. Then, the cells were permeabilized with 0.1% v/v Triton X-100 for 5 min before staining. Filamentous actin (F-actin) was stained with TRITC-phalloidin (1 μg/ml) for 30 min. Cell nuclei were stained with DAPI (4 μg/ml) for 5 min to evaluate the cell density in each condition. Fluorescence micrographs of stained samples were observed by a fluorescence microscope (DMi8, Leica). Based on the captured fluorescence micrographs, the density, spreading area and aspect ratio of cells under different culture conditions were measured by the software of ImageJ.

After 7 days of culture in microchannels, the static culture and single FSS stimulation samples were stained by BCIP/NBT alkaline phosphatase color development kit. Under the catalysis of alkaline phosphatase, BCIP is hydrolyzed to produce a highly reactive product, which reacts with nitroblue tetrazolium (NBT) to form the insoluble dark blue to blue-purple NBT-formazan. The darker the color is, the more alkaline phosphatase (ALP) is expressed by cells. All of the stained samples were observed by an inverted microscope (DMi8, Leica).

### Gene expression evaluation

Reverse transcriptase-polymerase chain reaction (RT-PCR) was applied to measure the expression of osteogenic related genes. After 7 days of culture in the microchannels, the total RNA of all samples (static culture, single FSS stimulation and compound stimulation) was isolated by the MagneSil^@^ Total RNA mini isolation system (Promega) through recommended procedures [41]. cDNA synthesis and fluorescence quantitative testing were carried out by Shanghai Daixuan Biotechnology Co., Ltd. Data were analyzed by using the 2^-ΔΔCt^ method, and *β*-actin was chosen as the housekeeping gene. The primers used in this study are listed in Table 1.

**Table 1.** RT-PCR primer sequences of the tested genes

	Forward primer (5′–3′)	Reverse primer (5′–3′)
OPN	CGCATTACAGCAAACACTCAG	GTCATCGTCGTCGTCATCAT
Col I	TTAACAAGGGAGGAGAGAGTG	GGAGGGTTTCAGAAGAGAGA
*β*-Actin	CCTCTATGCCAACACAGT	AGCCACCAATCCACACAG

### Statistical analysis

All of the statistical data were presented by mean value ± standard deviation, and *n* = 3 for each group, unless otherwise indicated. One-way ANOVA analysis was applied to evaluate the differences between indicated groups. A difference was regarded as significance when *P* < 0.05 as usual.

## Results and discussion

### Compound stimulation platform for cell studies

**Figure 2.**
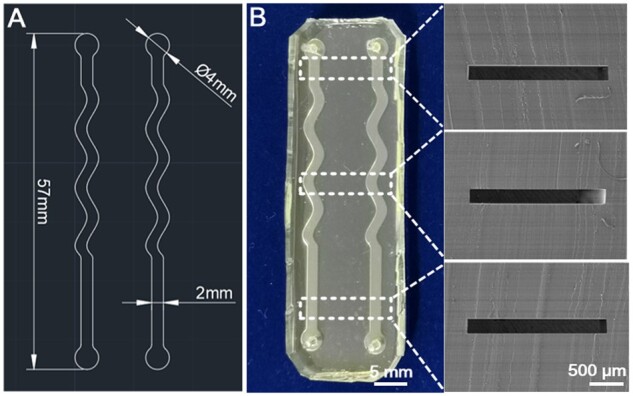
Characteristics of the fabricated microfluidic chip. (**A**) Original design drawing of the microfluidic channels by Auto-CAD. (**B**) Gross view of the fabricated microfluidic chip and corresponding SEM micrographs (section view) of the indicated locations of the microfluidic chip

The microchannel was designed with indicated curve features to increase the investigation region at some extent so that more cells could be observed, as shown in Fig. 2A. The height and width of the microchannel were designed to be about 200 μm and 2 mm, respectively. A typical PDMS microfluidic chip fabricated in this study was presented in Fig. 2B. As shown in the section view of the microchannel (SEM micrographs, the right part of Fig. 2B), the PDMS channels have high fidelity. Similar results could be obtained from the morphological features of the microchannel detected by a scanning white-light interferometry profilometer (see Supplementary Fig. S1).

As the culture medium flowing through the microchannel with varied perfusion rates, different FSS will be applied to the cells cultured on the bottom of the channel. The FSS $\tau_{w}$ (Pa, 1 Pa = 10 dyn/cm^2^) in the microchannel could be calculated by the equation [64] $\tau_{w}=6\mu Q/(bh^{2})$, where μ is the viscosity of perfusion liquid (about 0.001 Pa·s for cell culture medium), Q (m^3^/s) is the velocity of perfusion liquid, b (m) is the width of the channel and h (m) is the height of the channel. Thus, the FSS could be easily adjusted by changing the perfusion velocity (Q) of culture medium through the perfusion pump.

It is important to make sure that the cells will not be detached from the substrate under perfusion condition when investigating the FSS effect on cell behaviors. In order to promote cell adhesion in the microchannel and hence increasing the range of investigated FSS, the inner surface of all the microchannels were incubated and coated with 25 μg/ml fibronectin solution for 12 h in the incubator. It indicated that after the initial 3 h perfusion in the microchannel, the cell density showed no significant difference between different perfusion conditions and the static control, as shown in Supplementary Fig. S2. This modification strategy successfully improved the upper limit of perfusion rate to more than 45 μl/min (corresponding FSS in this condition is about 0.56 dyn/cm^2^) in our study.

In addition to the mentioned microfluidic chip for cell culture and perfusion system for adjusting FSS intensity, the whole compound stimulation platform was consisted of another independent part, which is the ultrasound system, as schematically illustrated in Fig. 1. The ultrasound stimulation was directly applied to the bottom of the microchannel through the ultrasonic probe coated with ultrasonic coupling agent, which can be easily used to load or unload ultrasound stimulation to the above-mentioned cell culture system. The combination of microfluidic chip, perfusion and ultrasound system successfully created a compound stimulation platform suitable for cell studies. Furthermore, the intensity of the FSS and ultrasound could be easily and separately regulated by controlling the propulsion rate of injection pump and the power of ultrasound instrument.

### Single FSS stimulation on stem cell proliferation and osteogenesis

In order to better understand the role of FSS-ultrasound compound stimulation on stem cell behaviors, the effects of single FSS stimulation on the adhesion, proliferation and osteogenesis of BMSCs in growth medium were firstly investigated. In this study, three typical perfusion rates (5, 15 and 45 μl/min; 6 h per day) were chosen to stimulate the cultured stem cells. Corresponding FSS applied on the cultured cells were about 0.06, 0.19 and 0.56 dyn/cm^2^, respectively. The cells cultured under static condition were set as control.

**Figure 3.**
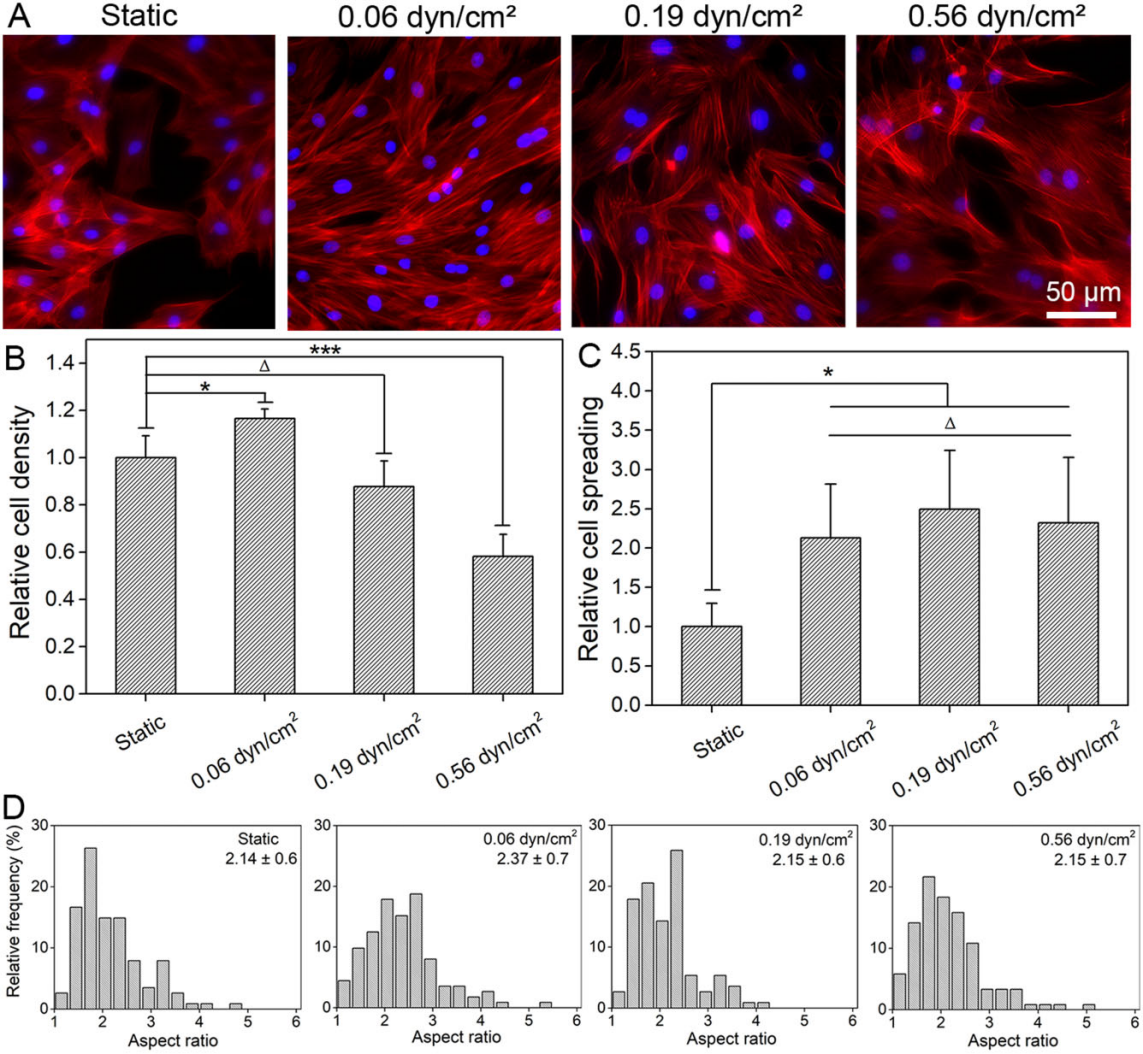
Cell adhesion under different FSS after 4 days of culture. (**A**) The fluorescence micrographs of BMSCs adhesion in microchannels under different FSS. Red: F-actin, blue: nuclei. (**B**)–(**D**) Statistical results of relative cell density, relative cell spreading and cell aspect ratio distribution of BMSCs under different FSS. Data on the top-right corner of (D) are mean ± SD of the corresponding aspect ratio of cells. ‘*’: *P* < 0.05, ‘***’: *P* < 0.001, ‘Δ’: *P* > 0.05

After 4 days of culture under different single FSS stimulations, cell cytoskeleton (F-actin) and nuclei were both stained. Typical fluorescence micrographs of different groups were shown in Fig. 3A. Through statistical analysis of the fluorescence micrographs from typical regions, results of relative cell density (see Fig. 3B), relative cell spreading (see Fig. 3C) and distribution of cell aspect ratio (see Fig. 3D) under indicated FSS stimulation were obtained.

Compared with the static control, the cell density under 0.06 dyn/cm^2^ FSS condition is much higher, as clearly shown in Fig. 3A and B, which indicated that proper FSS (0.06 dyn/cm^2^) stimulation could effectively promote BMSCs proliferation. However, as the intensity of FSS further increased, cell proliferation was gradually reduced, and even seriously inhibited under the condition of 0.56 dyn/cm^2^. It is probably because that the cell viability would be seriously reduced under higher FSS intensity. Similar inhibition of cell proliferation by higher FSS stimulation have also been reported by other literatures [65, 66].

In addition, the cell spreading under tested FSS stimulations were all significantly increased when compared with the static culture condition (see Fig. 3C), while there was no significant difference between varied FSS stimulations. This is probably due to the fact that cells intend to increase their spreading area for resisting the shear force generated by the flow of culture medium. Some related researches have also confirmed that cell cytoskeleton will respond to the internal or external physical cues, thereby guiding the cell to regulate its own adhesion or other behaviors to adapt corresponding microenvironment [67].

Cell aspect ratios in different culture groups were also carefully measured. Compared with the static control, the evaluated FSS stimulations have increased cell aspect ratio at some extent, as shown in Fig. 3D. Liu *et al*. [68] have reported that when cells were subjected to FSS, cell cytoskeleton will be rearranged, and hence changed their morphological features. In this study, the cell aspect ratio increased slightly under higher FSS conditions (0.19 and 0.56 dyn/cm^2^), while increased obviously under 0.06 dyn/cm^2^ FSS stimulation, as shown in Fig. 3D. This is probably due to the fact that the largest cell number has been presented under this suitable FSS condition (0.06 dyn/cm^2^) due to its optimal condition for cell proliferation (see Fig. 3B), resulting in obvious intercellular squeezing to further promote the elongation of stem cells.

In order to investigate the effect of FSS stimulation on the osteogenic differentiation of stem cells, ALP, a specific protein expressed in the early stage of osteogenesis [69], was stained after 7 days of culture under different single FSS stimulations. Moreover, osteogenic specific gene expression, such as osteopontin (OPN) and college I (Col I) were also evaluated. OPN, a non-collagen bone matrix protein, is a phosphorylated bone matrix salivation protein that plays an important role in cell adhesion and biomineralization [70]. Col I is the main collagen type in bone tissue [70].

**Figure 4.**
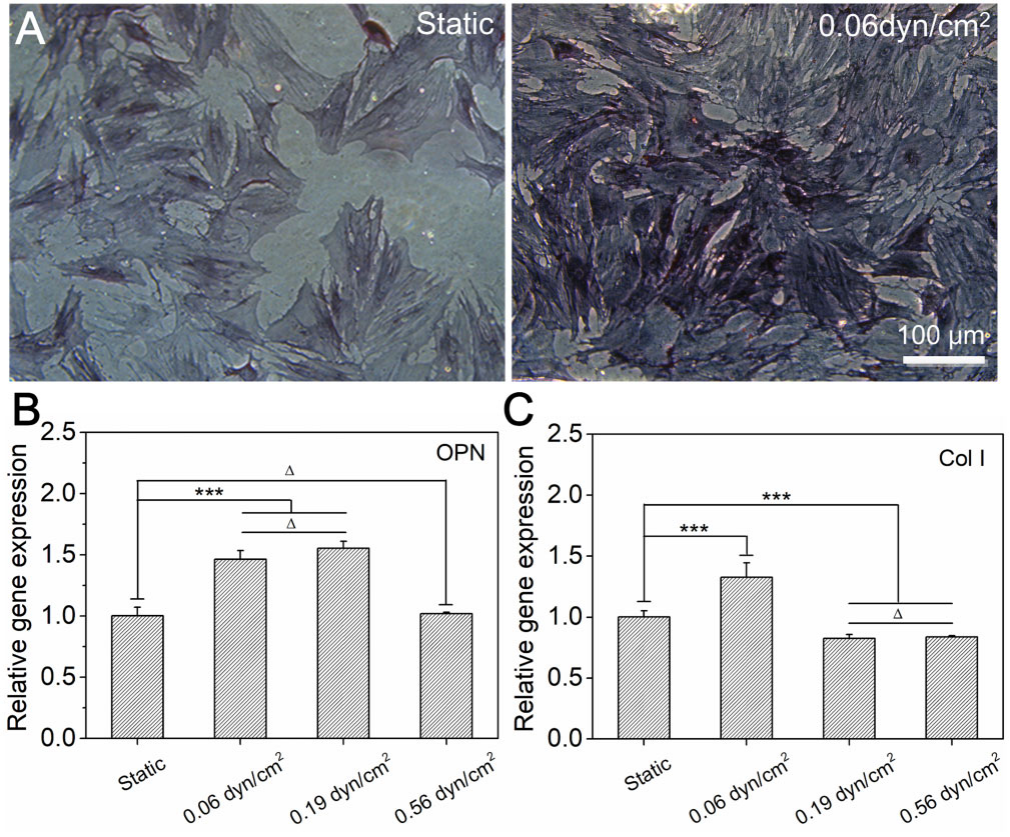
Osteogenesis of BMSCs under different FSS after 7 days of culture in growth medium. (**A**) Phase contrast micrographs of cells under different FSS. Before observation, the ALP (an indicator of osteoblasts) was stained by BCIP/NBT alkaline phosphatase color development kit. (**B**) and (**C**) Statistical results of relative gene (OPN, col I) expression of BMSCs under varied FSS after 7 days of culture. ‘***’: *P* < 0.001, ‘Δ’: *P* > 0.05

The staining results of ALP and statistical results of specific genes expression were presented in Fig. 4. The ALP staining of stem cells under static culture condition showed a light blue color. However, after the stimulation of an appropriate FSS (0.06 dyn/cm^2^), the expression of ALP was significantly increased, therefore presented a dark blue color (see Fig. 4A). In addition, the expressions of OPN and Col I were both significantly upregulated under this suitable FSS stimulation (0.06 dyn/cm^2^) when compared to static control, as shown in Fig. 4B and C. More specifically, as for the expression of OPN, the group of 0.19 dyn/cm^2^ FSS was almost similar to that of the 0.06 dyn/cm^2^ FSS (no significant difference), both significantly upregulated when compared to the static control, while the group of 0.56 dyn/cm^2^ FSS presented similar level to the static control. As for the expression of Col I, the groups with higher FSS stimulations (0.19 and 0.56 dyn/cm^2^) were both obviously downregulated when compared to the static control, while the group of 0.06 dyn/cm^2^ FSS was still significantly upregulated. Taken together, proper FSS stimulation (0.06 dyn/cm^2^) has obviously enhanced stem cell osteogenesis, as the intensity of FSS further increased, the expressions of osteogenic specific genes were gradually decreased.

For the difference of cell differentiation, on the one hand, stem cells presented the optimal proliferation ability under the condition of 0.06 dyn/cm^2^ FSS (see Fig. 3B) in our tested groups, and hence induced higher cell density and more cell–cell contact. On the other hand, the spreading area of stem cells under this condition was also significantly increased when compared to static control (see Fig. 3C). It has been reported that more cell–cell contact and larger cell spreading were both beneficial for the osteogenic differentiation of stem cells [40, 71–74]. That may be the possible reason for why the highest osteogenic characteristic gene expression happened under this suitable FSS stimulation (0.06 dyn/cm^2^). Under the stimulation of 0.19 dyn/cm^2^ FSS, the cell density and cell–cell contact were relatively lower than that of the 0.06 dyn/cm^2^ FSS group, while the spreading area of the cell was similar. Consequently, the expression of characteristic gene became lower. Under the stimulation of 0.56 dyn/cm^2^ FSS, cell proliferation was significantly inhibited (see Fig. 3B), the cell density and cell–cell contact were both obviously decreased under this condition and hence eventually led to a lowest expression of osteogenic specific genes.

### Compound stimulation of FSS plus ultrasound on stem cell proliferation and osteogenesis

Cells in the body are often exposed to a complex microenvironment with varied stimulations. Herein, FSS plus ultrasound stimulations were selected as a model to investigate the effects of compound stimulation on stem cell behaviors for the first time. Based on the evaluation of single FSS stimulation, it was found that both the proliferation and osteogenic differentiation could be significantly enhanced under the proper intensity of FSS stimulation (0.06 dyn/cm^2^, the perfusion condition is 5 μl/min). So, the perfusion condition was fixed as 5 μl/min to investigate the effects of FSS plus ultrasound stimulations on the adhesion, proliferation and differentiation of stem cells. The intensities of combined ultrasound in this study were 0.075, 0.5 and 1.75 W/cm^2^, and corresponding compound stimulation groups were denoted as I, II and III, respectively.

**Figure 5.**
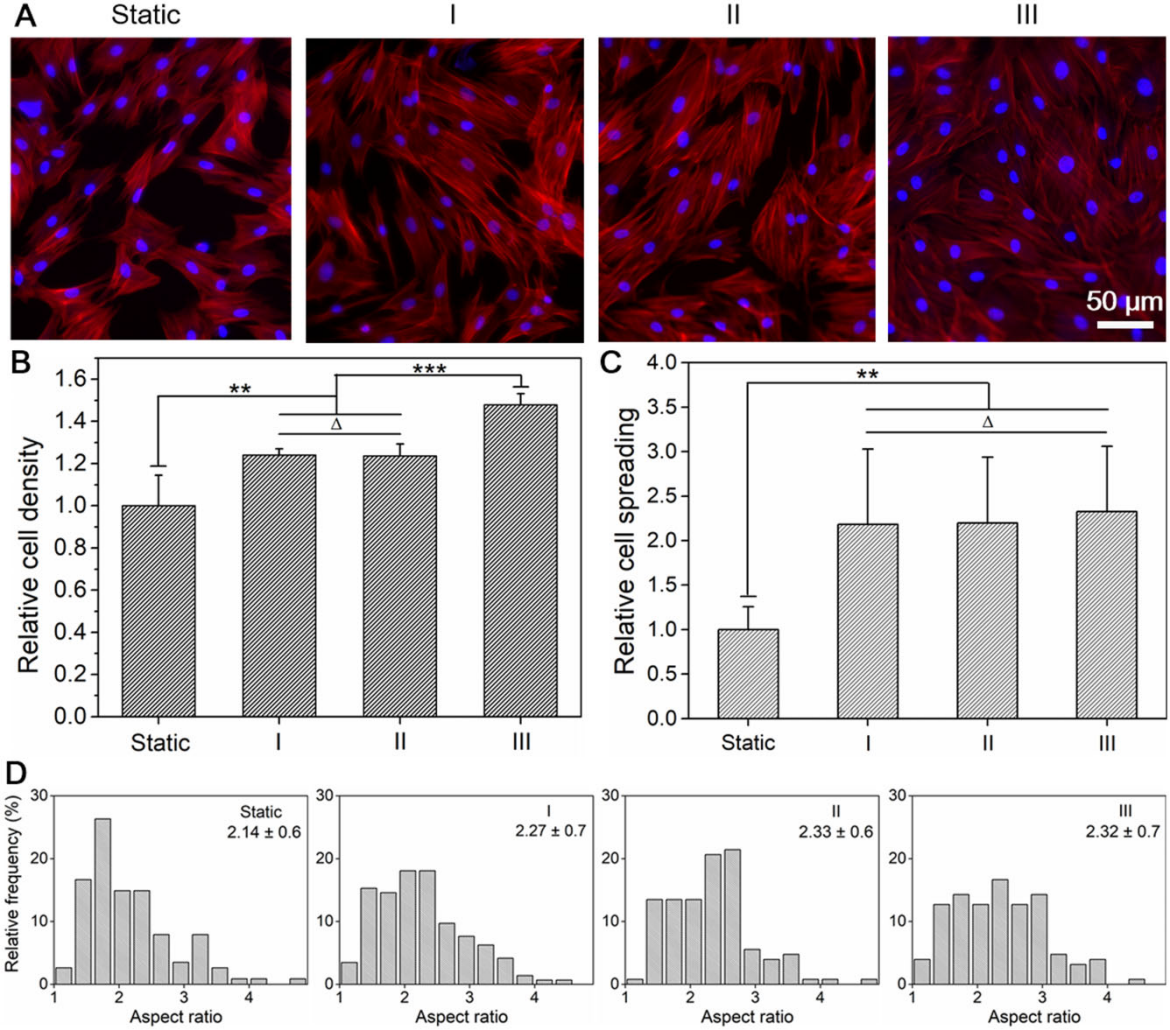
Cell adhesion under different ultrasound stimulations combined with the same FSS of 0.06 dyn/cm^2^ after 4 days of culture. (**A**) The fluorescence micrographs of BMSCs adhesion in microchannels with indicated compound stimulations. Static: without FSS and ultrasound, I: 0.075 W/cm^2^ ultrasound combined with 0.06 dyn/cm^2^ FSS, II: 0.5 W/cm^2^ ultrasound combined with 0.06 dyn/cm^2^ FSS, III: 1.75 W/cm^2^ ultrasound combined with 0.06 dyn/cm^2^ FSS. Red: F-actin, blue: nuclei. (**B**)–(**D**) Statistical results of relative cell density, relative cell spreading and cell aspect ratio distribution of BMSCs under different compound stimulations. Data on the top-right corner of (D) are mean ± SD of the aspect ratio of cells. ‘**’: *P* < 0.01, ‘***’: *P* < 0.001, ‘Δ’: *P* > 0.05

Typical fluorescence micrographs of stem cells and corresponding statistical results of cell density, cell spreading and cell aspect ratio distribution after 4 days of culture with compound stimulations were shown in Fig. 5. FSS plus ultrasound stimulation significantly promoted the proliferation of stem cells when compared to static control. Moreover, compared with the results of single FSS stimulation (0.06 dyn/cm^2^), the combination of ultrasound stimulation further increased cell proliferation as the ultrasound intensity increased, as shown in Supplementary Fig. S3A. That may be the possible reason for that the ultrasound stimulation could promote the healing of wounds or defects reported before [59].

According to the statistical results of cell morphological features, the cell spreading areas under compound stimulation were all significantly higher than static control, while no significant difference was found between each compound stimulation group (see Fig. 5C). Besides, when compared to single FSS stimulation (0.06 dyn/cm^2^), cell spreading showed no further significant changes under the compound stimulation (see Supplementary Fig. S3B). Taken together, it indicates that the FSS stimulation may be the dominant factor for the increase of cell spreading under the stimulation of FSS plus ultrasound, while ultrasound stimulation has no obvious effect on cell spreading.

The distribution diagrams of cell aspect ratio under different compound stimulations were shown in Fig. 5D. When compared to the static control, the average aspect ratio of the cells was all increased under the compound stimulation. This is probably because of the fact that compound stimulation obviously enhanced the proliferation of stem cell (see Fig. 5B) and corresponding intercellular squeezing under the condition of higher cell density elongates cell aspect ratio.

**Figure 6.**
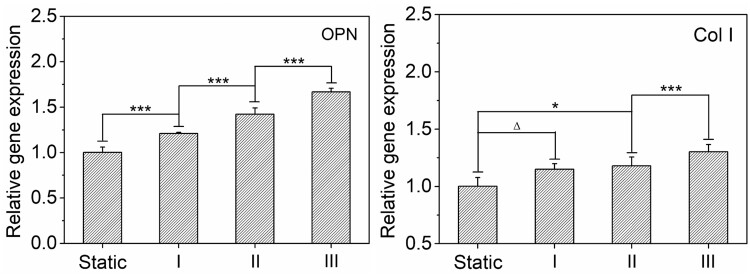
Statistical results of relative gene expression of BMSCs under varied ultrasound stimulations combined with the same FSS of 0.06 dyn/cm^2^ after 7 days of culture. ‘*’: *P* < 0.05, ‘***’: *P* < 0.001, ‘Δ’: *P* > 0.05

In order to reveal the effect of compound stimulation on osteogenic differentiation of stem cells, the expressions of OPN and Col I were both characterized after 7 days of culture in growth medium under FSS plus ultrasound stimulations. Relative gene expressions were illustrated in Fig. 6. When compared to the static control, both OPN and Col I were gradually upregulated as the combined ultrasound intensity increased. Furthermore, comparing with the results of single FSS stimulation, the expression of osteogenic characteristic genes (OPN and Col I) was globally decreased when the combined ultrasound intensity is lower (0.075 or 0.5 W/cm^2^), as shown in Supplementary Fig. S4. However, it was then globally increased higher than single FSS stimulation when the combined ultrasound intensity further increased to 1.75 W/cm^2^.

Compared with the single FSS stimulation, the cell density and cell–cell contact were increased as the ultrasound intensity increased under compound stimulation, while cell spreading area presented no obvious changes. In addition, when compared to the static group, both the cell spreading and cell–cell contact were increased in the compound stimulation groups. From the perspective of the cell–cell contact and cell spreading effects on BMSCs osteogenesis reported before [40], the osteogenic differentiation should be promoted under compound stimulation when compared to single FSS stimulation or static culture own to the increased cell–cell contact and cell spreading. However, the expressions of OPN and Col I were even decreased to some extent compared to single FSS stimulation and similar to that of the static group under the conditions with relative lower ultrasound intensity (see Supplementary Fig. S4). Only when the combined ultrasound intensity further increased to 1.75 W/cm^2^, the gene expression could be improved. Therefore, combined ultrasound stimulation in this study may afford another independent factor (different from cell spreading and cell–cell contact) for comprehensively regulating the osteogenic differentiation of stem cells. Under relatively lower ultrasound intensity, it will inhibit the cell osteogenesis to a certain extent, while at a relatively higher stimulation intensity it will act as a promoter for stem cell osteogenesis.

## Conclusions

In summary, we have constructed a compound stimulation (FSS plus ultrasound) platform suitable for cell studies based on a microfluidic chip. Compared to static culture condition, a suitable intensity (0.06 dyn/cm^2^) of single FSS stimulation was found to be beneficial both for cell proliferation and osteogenesis, while this kind of enhancing effect gradually attenuated or even evolved into negative effect as the FSS intensity further increased. Based on this suitable FSS stimulation (0.06 dyn/cm^2^), stem cell proliferation was further enhanced with the increase in the intensity of combined ultrasound stimulation. In addition, stem cell osteogenic differentiation could also be further promoted under the compound stimulation with a higher ultrasound intensity. Corresponding results illustrated that both the FSS and ultrasound stimulations could profoundly regulate stem cell behaviors in an intensity-dependent manner. Under proper combination strategies, both the proliferation and differentiation of stem cell could be obviously enhanced. The construction of compound stimulation platform and corresponding revealed effects on stem cell behaviors are valuable for guiding the design of novel cell bioreactors. Furthermore, these findings might also be meaningful for the prevention and treatment of bone tissue related diseases or defects.

## Supplementary data

Supplementary data are available at *REGBIO* online.

## Supplementary Material

rbab066_Supplementary_Data
