# Supplementary material for: Effects of compound stimulation of fluid shear stress plus ultrasound on stem cell proliferation and osteogenesis
Source: Regen Biomater. 2021 Nov 18;8(6):rbab066. doi: 10.1093/rb/rbab066 (PMC8634505; doi:10.1093/rb/rbab066)
Supplement: rbab066_Supplementary_Data [file rbab066_supplementary_data.docx]

Supporting Information for

**Effects of compound stimulation of fluid shear stress plus ultrasound on stem cell proliferation and osteogenesis**

Lingzhi Jing ^1^, Suna Fan ^1,2^, Xiang Yao ^1,^*, Yaopeng Zhang ^1,2,^

^1^. State Key Laboratory for Modification of Chemical Fibers and Polymer Materials, Shanghai Engineering Research Center of Nano-Biomaterials and Regenerative Medicine, College of Materials Science and Engineering, Donghua University, Shanghai 201620, People’s Republic of China

^2^. Jinan Jinquan Bio-Technology Co. Ltd., Jinan 250101, People’s Republic of China

* Corresponding author

E-mail: yaoxiang@dhu.edu.cn (X. Yao)


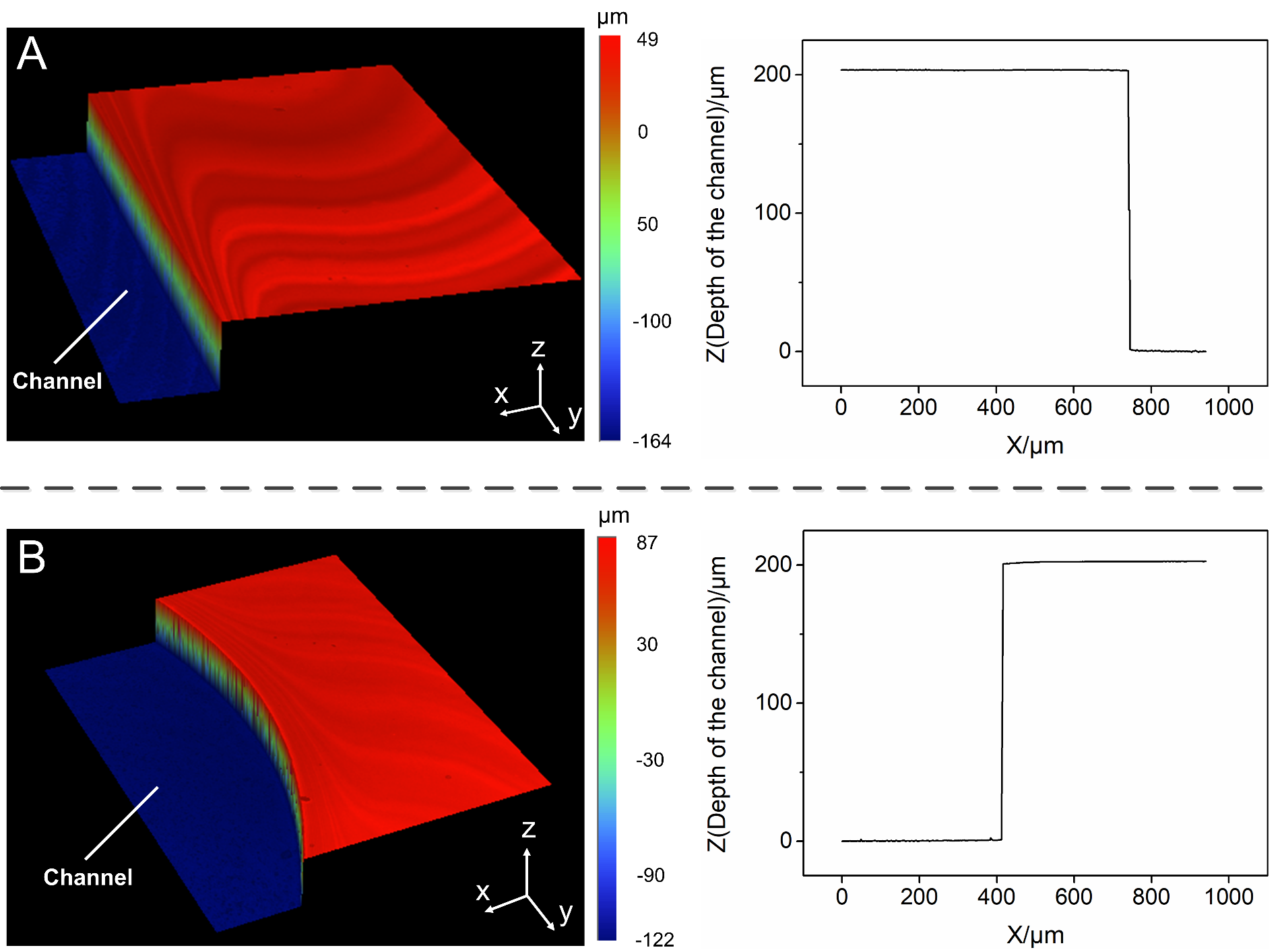


**Figure S1.** Morphological features of the microchannel measured by a scanning white-light interferometry profilometer. (A) Near the outlet part of microchannel, (B) Near the middle part of microchannel.


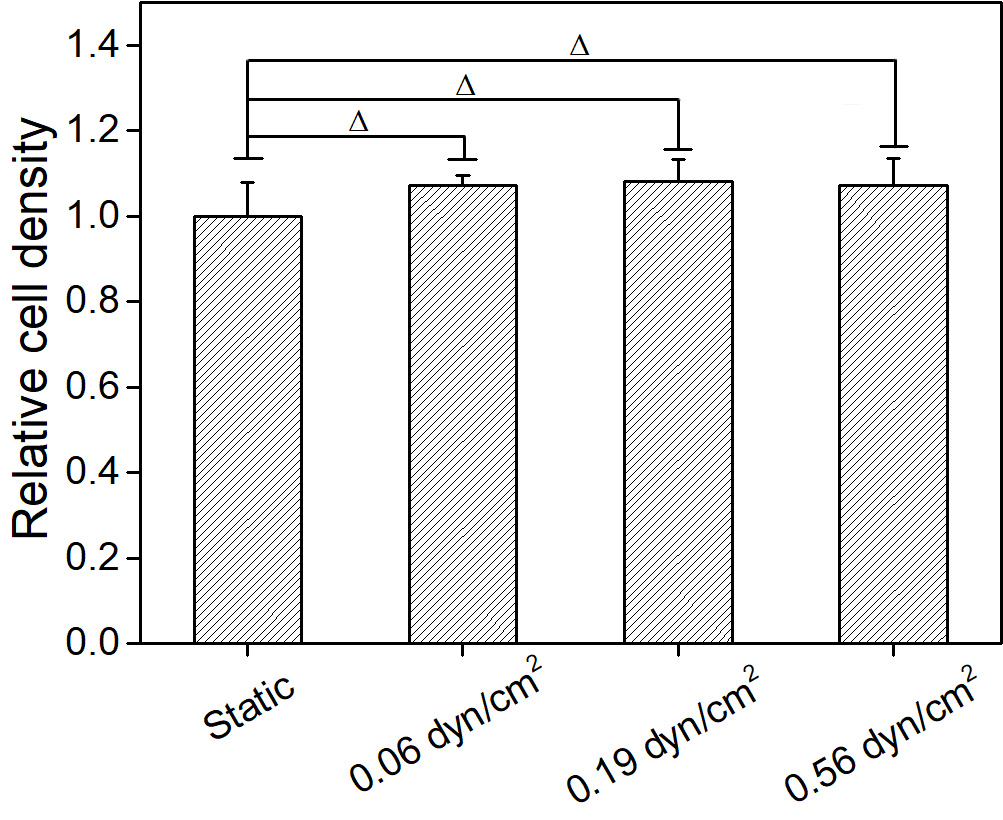


**Figure. S2.** Statistical results of relative cell density under different FSS after the initial 3 h perfusion. “Δ”: *p* > 0.05.


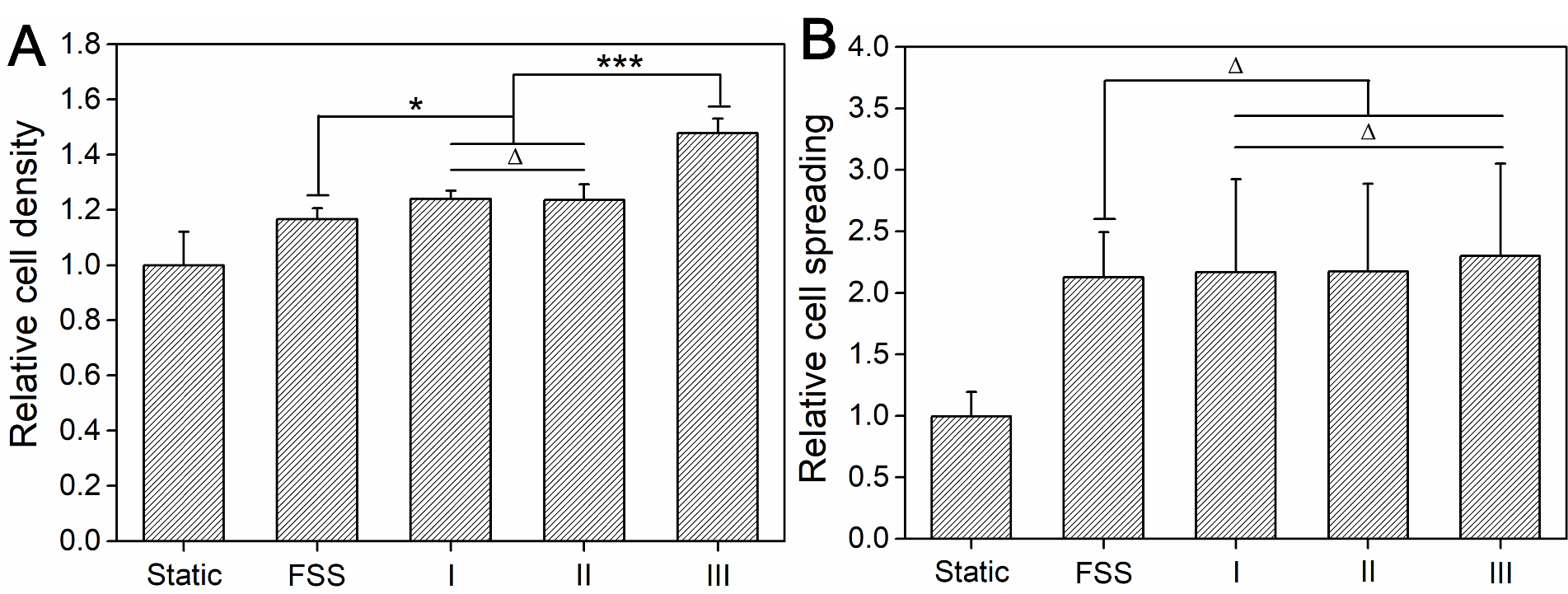


**Figure. S3.** Cell adhesion under static, single FSS stimulation (0.06 dyn/cm^2^) and compound stimulation (different ultrasound stimulations combined with the same FSS of 0.06 dyn/cm^2^) conditions after 4 days of culture. (A) Statistical results of relative cell density of BMSCs under varied culture conditions. This figure combined the data of Fig. 3B and Fig. 5B. (B) Statistical results of relative cell spreading of BMSCs under varied culture conditions. This figure combined the data of Fig. 3C and Fig. 5C. “*”: *p* < 0.05, “***”: *p* < 0.001, “Δ”: *p* > 0.05.


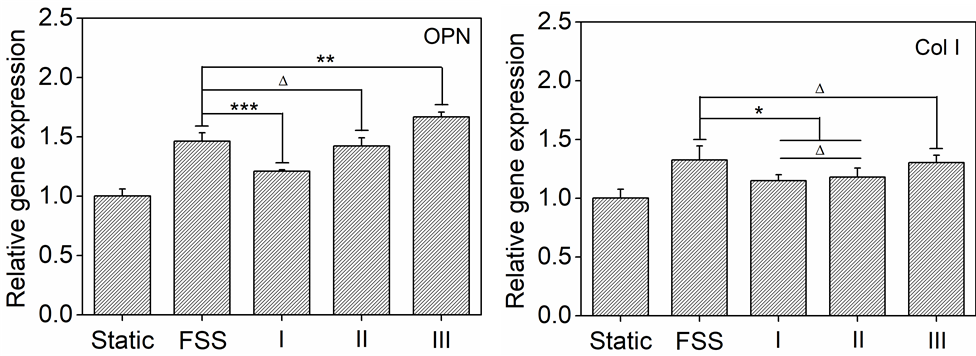


**Figure. S4.** Statistical results of relative gene expression of BMSCs under static, single FSS stimulation (0.06 dyn/cm^2^) and compound stimulation (different ultrasound stimulations combined with the same FSS of 0.06 dyn/cm^2^) conditions after 7 days of culture. This figure combined the data of Fig. 4B-C and Fig. 6. “*”: *p* < 0.05, “**”: *p* < 0.01, “***”: *p* < 0.001, “Δ”: *p* > 0.05.
